# Epigenome-Wide Association Study (EWAS) of Blood Lipids in Healthy Population from STANISLAS Family Study (SFS)

**DOI:** 10.3390/ijms20051014

**Published:** 2019-02-26

**Authors:** Ting Xie, Vesna Gorenjak, Maria G. Stathopoulou, Sébastien Dadé, Eirini Marouli, Christine Masson, Helena Murray, John Lamont, Peter Fitzgerald, Panagiotis Deloukas, Sophie Visvikis-Siest

**Affiliations:** 1 Université de Lorraine, Inserm, IGE-PCV, 54000 Nancy, France; ting.xie@inserm.fr (T.X.); gorenjak.vesna@gmail.com (V.G.); maria.stathopoulou@inserm.fr (M.G.S.); sebastien.dade@univ-lorraine.fr (S.D.); christine.masson@univ-lorraine.fr (C.M.); 2 Queen Mary University of London, E1 4NS London, UK; e.marouli@qmul.ac.uk (E.M.); p.deloukas@qmul.ac.uk (P.D.); 3 Randox Laboratories Ltd, Crumlin, BT29 4XL County Antrim, UK; helena.murray@randox.com (H.M.); john.lamont@randox.com (J.L.); peter.fitzgerald@randox.com (P.F.); 4 Department of Internal Medicine and Geriatrics, CHU Nancy-Brabois, 54000 Nancy, France

**Keywords:** genetics, gene Expression/polymorphisms, lipids, adipose tissue, dyslipidemias, EWAS, DNA methylation, epigenetics

## Abstract

Epigenome-Wide Association Studies (EWAS) are furthering our knowledge of epigenetic modifications involved in the regulation of lipids’ metabolism. Furthermore, epigenetic patterns associated with lipid levels may play an important role in predicting the occurrence of cardiovascular events. To further investigate the relationship between methylation status and lipids, we performed an EWAS in 211 individuals from the STANISLAS Family study (SFS). Methylation at two CpG sites (*PRKAG2*; *p* = 1.39 × 10^−8^; *KREMEN2*; *p* = 5.75 × 10^−9^) were significantly associated with lipidomic profiles. Replication was sought in adipose tissue where one probe, cg08897188, was found to be nominally significant (*KREMEN2*; *p* = 0.0196). These results could provide new insight in the mechanisms underlying cardiovascular diseases and contribute to new therapeutic interventions.

## 1. Introduction

Our health and well-being depend on diverse interactions between clinical, biological, environmental and lifestyle factors, which, all together, are involved in complex pathological mechanisms that may influence the disease susceptibility, development and prognosis of the treatment [1]. Dysregulated lipid levels, commonly referred to as dyslipidemia, are one of the leading risk factors for cardiovascular diseases (CVD), but also influence other chronic conditions such as type-2 diabetes mellitus [2]. Type-2 diabetes patients and individuals suffering from infectious diseases (e.g., AIDS and tuberculosis) were characterized by abnormal profiles of high triglycerides (TG), low levels of high density lipoprotein (HDL), high serum levels of very low density lipoproteins (VLDL) and increased LDL [2]. Cholesterol is one of the first biomarkers used in routine clinical diagnostics, and it can be well regulated with common treatment in patients with abnormal lipid profiles [3]. However, TG are now becoming the subject of new investigations in order to improve diagnosis and, potentially, in the prevention and treatment of CVD. Their predictive value for the onset of the pathology is considered to be even higher than that of cholesterol [4,5]. With further research, potential new medications for the normalization of TG levels could be developed and used together with statins to help further reduce risk of chronic diseases. CVD is the leading cause of premature mortality and morbidity in occidental countries, and is an essential contributing factor in the rise of health care costs [6]. The study of factors affecting lipids’ levels is an important field in the fight against CVD and other morbidities.

Lipid traits were found to be highly heritable, especially plasma cholesterol and TG concentrations (55–77%). However, changes in our environment can make modest, but clinically important changes regarding common risk factors [7]. The genetics of blood lipids have been assessed in a large genome-wide association study (GWAS), where as many as 157 lipid-associated loci were discovered (8), accounting for more than 12% of the total variance [8]. Strong associations were found between variants near *LIPC* gene with plasmalogen levels (*p* < 10^−40^), and variants near *ABCA1* gene with sphingomyelin levels (*p* < 10^−5^) [9]. These variants were previously associated with cardiovascular and metabolic traits including coronary artery disease, type 2 diabetes, blood pressure, waist/hip ratio, and body mass index, showing that even variants with small effect size can expose new pathways and therapeutic targets [9,10].

Epigenome-wide association studies (EWAS) are the epigenetic equivalent of GWAS, giving us information about associations between epigenomic perturbations and traits related to human diseases [11]. They allow us to assess the environmental impact on genetic regulation. With progress in epigenetics, new insights on disease mechanisms are possible through the exploration of the DNA accessibility and chromatin structure, and are enabling us to explain the regulatory mechanisms of gene expression. Epigenetic variations could explain parts of missing heritability of chronic diseases that have not yet been elucidated by GWAS [12]. The most common mechanism of epigenetic patterns is DNA methylation, which is the forming of 5-methylcytosine on the CpG (cytosine-phosphate-guanine) site of the genome; it normally results in silencing of the gene encoded in the sequence [13].

Recent EWAS studies are furthering the current knowledge of epigenetic modifications by investigating patterns associated with methylation at CpG sites in relation to the regulation of lipids. Thirty-three CpG sites were discovered and replicated in recent studies, located in genes associated with TG and HDL cholesterol, such as a reverse cholesterol transporter (*ABCG1; p* = 7.2 × 10^−28^). Pathway analysis showed the involvement of CpG sites in lipid, sterol, and cholesterol metabolic and biosynthetic processes [14]. Also, a differentially methylated locus was associated with CVD events (hazard ratio per SD increment, 1.38; 95% confidence interval, 1.15–1.66; *p* = 0.0007). These findings demonstrate an important role of epigenetic patterns in lipid metabolism and in predicting the occurrence of CVD events [14]. Several epigenetically regulated genes were shown to alter lipid levels, including carnitine palmitoyltransferase 1A *(CPT1A)*, ATP-binding cassette sub-family G member 1 *(ABCG1)*, sterol regulatory element binding transcription factor 1 *(SREBF1)*, troponin T1 (*TNNT1)*, microRNA 33b (*MIR33B)*, NFAIP3 interacting protein 3 *(TNIP)* and 24-dehydrocholesterol reductase (*DHCR24)* [14,15,16,17,18]. A differential methylation observed in EWAS was found to be a consequence, rather than a cause, of blood lipid abnormalities, since differential methylation was induced by TG, LDL and HDL, whereas there was no effect of DNA methylation on lipid levels in prime circulating immune cells [19].

All the aforementioned studies were performed on subjects suffering from cardiovascular condition. Regulation of lipid traits can differ in healthy population and new variants involved in gene regulation pathways can be discovered, which can contribute to a better understanding of the complex mechanisms involved in lipid metabolism. The aim of the current study was to fill this knowledge gap and analyze the association of epigenetic methylation patterns with lipid phenotypes (TG, cholesterol, LDL and HDL) in a healthy population and to expand the recent knowledge in the epigenetics of lipid profiles.

## 2. Results

### 2.1. Associations between Genome-Wide DNA Methylation and Lipid Levels in Whole Blood

Two probes demonstrated statistically significant levels of association with TG concentrations, including individual probes in *PRKAG2* on chromosome 7 (7q36.1), and *KREMEN2* on chromosome 16 (16p13.3) (Table 1). No significant associations were found between DNA methylation and other assessed lipid phenotypes (*p* < 0.05).

### 2.2. Bioinformatics Analysis

**Figure 1:**
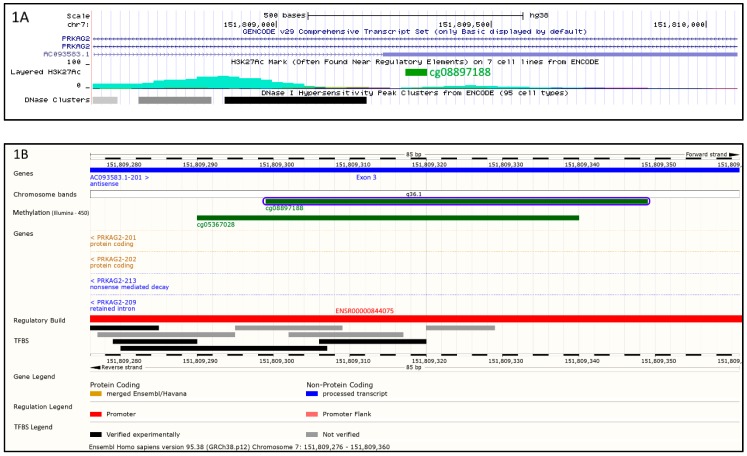
Environment of cg08897188 probe. As depicted by Figure 1A,B, cg08897188 (in green) is located on chromosome 7q36.1 within intron of *PRKAG2* gene of the forward strand and within exon 3 of the *AC093583.1* non-coding transcript in the opposite strand. (**A**) shows that cg08897188 is surrounded by regulatory elements indicated by acetylation of histone 3 on lysine 27 (H3K27Ac peaks, in turquoise). Moreover, as indicated by DNase I hypersensitivity peak clusters (black and grey rectangles), this region has also an accessible chromatin zone, indicating a transcriptional activity. (**B**) confirms that cg08897188 is located within a regulatory zone (promoter ENSR00000844075, in red) with clearly identified transcription factor binding sites (black and grey rectangles).

The cg08897188 probe maps on chromosome 7q36.1 and is located both within an intron of the *PRKAG2* gene and in the opposite strand within the *AC093583.1* non-coding transcript (Figure 1A). This gene has 2 splice variants: AC093583.1-201 (LOC644090) and AC093583.1-202. Cg08897188 is located in exon 3 of LOC644090 and in silico analysis showed the presence of transcription factor binding sites (Figure 1B).

**Figure 2:**
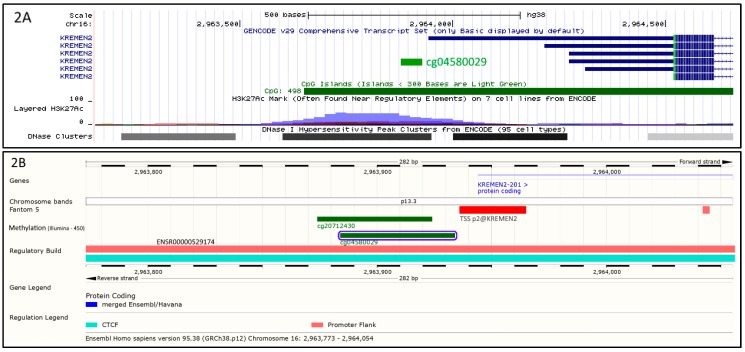
Environment of cg04580029 probe. As depicted by Figure 2A,B, cg04580029 (in green) is located on the chromosome 16p13.3 upstream of *KREMEN2* gene. (**A**) shows that cg04580029 is located in a regulatory element, indicated by acetylation of histone 3 on lysine 27 (H3K27Ac peak, in violet). Furthermore, DNase I hypersensitivity peak clusters (black and grey rectangles) reveal the presence of an accessible chromatin zone which is the sign of a transcriptional activity. (**B**) confirms that cg04580029 is located within a regulatory zone (promoter flank ENSR00000529174, in light red) and shows in addition that 3 base pairs away is located the transcription start site of *KREMEN2* gene, p2@KREMEN2.

The cg04580029 on the chromosome 16p13.3 is located in the promoter flank of the *KREMEN2* gene (ENSR00000529174) (Figure 2). The cg04580029 probe fully overlaps a DNase I hypersensitivity site and a H3K27Ac histone peak (Figure 2A). Further analysis showed that cg04580029 is allocated 3 base pairs away of the transcription start site of *KREMEN2* gene, p2@KREMEN2 (Figure 2B).

**Figure 3:**
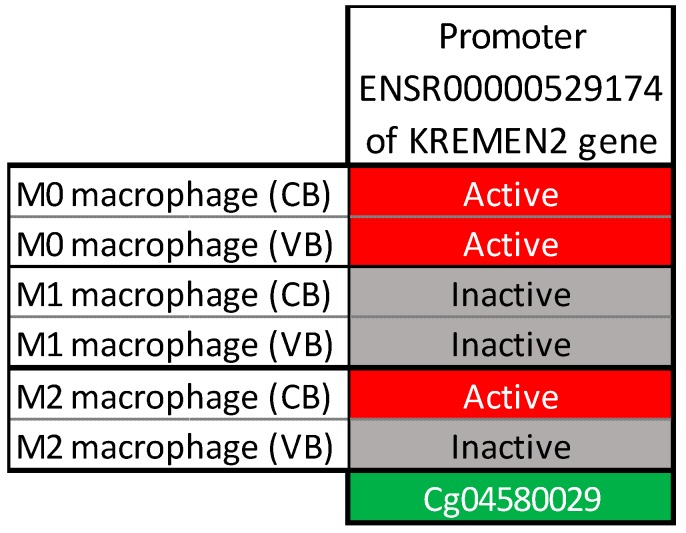
Regulatory status of the ENSR00000529174 promoter according to the macrophage cell type (CB: cord blood; VB: vein blood).

Analysis of the regulatory activity of the ENSR00000529174 promoter, where the cg04580029 is involved, shows that, depending on the level of macrophage differentiation, the promoter is either in an active or inactive state (Figure 3).

### 2.3. Associations between Genetic Variants and Methylation Sites

DNA sequence variation could influence methylation levels, thus we selected SNPs previously associated with TG levels in GWAS studies located in the same chromosomes of the identified methylation sites (Table 1) to test their associations with methylation levels. Sixteen polymorphisms have been identified in the chromosomes 7 and 16, which have previously been associated with TG levels. Cut off point for significance was set to 0.003, but no significant *cis* methylation QTL was detected.

### 2.4. Replication of the Identified CpGs Associations with Lipids Levels in Adipose Tissue

The associations of the 2 probes with TG were tested in adipose tissue and the results are presented in Table 2. One of these associations was also identified, however, in nominal significance level (cg04580029).

## 3. Discussion

This study confirmed the involvement of DNA methylation in the regulation of TG levels through possible metabolic pathways that are associated with the development of chronic diseases. Knowing how the complex processes of methylation can impact disease development might highlight new targets for treatment and contribute to the improvement of personalized strategies of patient care. We reported two new CpG sites, cg08897188 and cg04580029 that are associated with TG levels in a healthy population and replicated the cg04580029 in the adipose tissue.

Adipose tissue is composed of adipocytes in its parenchymal part and also contains a stromal vascular fraction (STV) for its mesenchymal part. Thus, STV includes preadipocytes, fibroblasts, vascular endothelial cells and a variety of immune cells such as macrophages. It is therefore not surprising to note that the promoters of these cell-types are active and can be regulated by CpG methylation (Figure 3).

**Figure 4:**
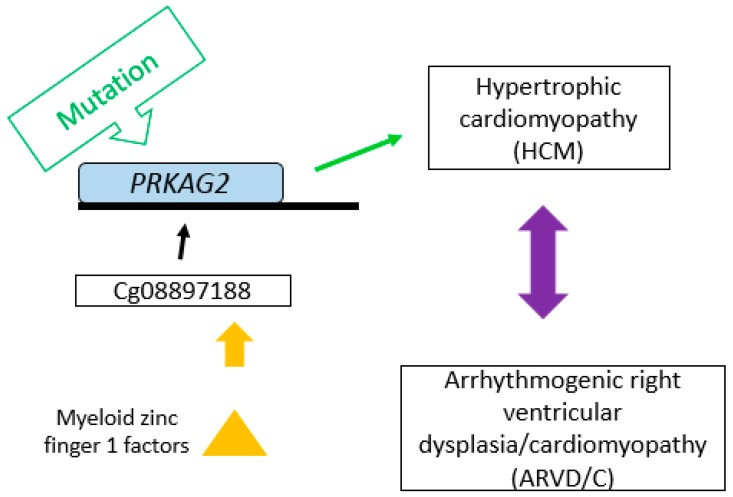
Possible mechanism of association of cg08897188 with CVD. Cg08897188 is involved in regulation of the *PRKAG2* gene. Mutation of *PRKAG2* can cause HCM or ARVD/C. Cg08897188 has a specific region able to bind myeloid zinc finger 1 factor, which is also associated with ARVD/C.

The cg08897188 probe is located closed to the distal end of a DNase I hypersensitive site and a H3K27Ac histone peak which is a mark for active transcription. As shown in Figure 1A, the proximity of cg08897188 to the H3K27Ac histone peak implies that it may be involved in the transcriptional regulation of this region. The region harbors the *PRKAG2* gene, coding for a protein involved in the regulation of fatty acid metabolic process [20] and fatty acid oxidation [21], as well as an EST-based antisense non-coding transcript LOC644090 (AC093583.1-201) on the opposite strand (Figure 1A,B). Therefore, the effect of cg08897188 could be exerted either via enhancing the transcription of LOC644090 which in turn down-regulates *PRKAG2* expression or directly modulating *PRKAG2* expression. PRKAG2 is an AMP/ATP-binding subunit of AMP-activated protein kinase (AMPK), an energy sensor protein kinase that plays a key role in regulating cellular energy metabolism and therefore may be implicated in fatty acid oxidation and modulation of TG level. The mutations in *PRKAG2* were first related to familial hypertrophic cardiomyopathy (HCM) [22], yet Arad et al. revealed that the *PRKAG2* mutations affect metabolic cardiomyopathy rather than HCM [23]. Finally, the mutation of *PRKAG2* is known to cause the glycogen-storage cardiomyopathy that resembles HCM but is distinguished by electrophysiological abnormalities [24]. Our results might support these findings, since the probe cg08897188 has a specific region that is able to bind MZF1 (Myeloid zinc finger 1 factors), which are associated with arrhythmogenic right ventricular dysplasia or cardiomyopathy (ARVD/C) with a similarity score of 0.992 [25]. There may be an association between the methylation profile that affects TG levels and a heritable heart muscle disease (ARVD) (Figure 4). However, further experiments are required to confirm this assumption and to make further progress in the investigation of the disease mechanisms.

The cg04580029 is located in the promoter flank of *KREMEN2* gene, only 3 bp away of the TSS (p2@KREMEN2—Figure 2B). The observed overlap with a H3K27Ac histone peak which is a mark for active transcription, implies that modulation of methylation levels at cg04580029 will directly impact *KREMEN2* expression. It is well known that LRP6 (low-density lipoprotein-related receptors 6) is a co-receptor of WNT that transmits the canonical Wnt/β-catenin signaling cascade, which has been associated with many diseases including Alzheimer’s disease [26] and coronary artery disease (CAD) [27,28]. In the canonical way, extracellular Wnt protein binds to a complex transmembrane receptor composed of Frizzled (Fz) and LRP (LDL-related protein) allowing to recruit intracellular Dishevelled (Dsh) protein. Dsh, in turn, recruit GSK3 (glycogen synthase kinase) protein that will not be able to phosphorylate β-catenin, which avoids to be degraded by proteasome. Thus, β-catenin can act as a transcription factor and stimulate gene expression. Due to its importance, this signaling pathway is closely controlled, in particular by an extracellular inhibitor called Dickkopf (DKK), which interacts with both the LRP5/6 co-receptor and the KREMEN1/2 proteins, and thus prevents Wnt dimerization of the LRP and Fz co-receptors (39). KREMEN2 protein prevents Wnt dimerization of the co-receptors LRP and Fz. Thus, a regulation of KREMEN2 via cg04580029 methylation near transcription start site might remove inhibition pathway of Wnt and in turn, β-catenin could stimulate specific genes expression leading to modulate TG level. Interestingly, our results found that the KREMEN2-related CpG site cg04580029 was significantly associated with TG levels in a healthy population. Therefore, our results suggest that KREMEN2 could be proposed as a link between TG and Alzheimer’s disease and CAD through methylation profiles. Furthermore, this association was also present in the adipose tissue, however at a nominal level of significance.

TG-related differential methylation has previously been described in several genes [14,18]. Methylation on *ABCG1* locus, associated with expression of a gene involved in reverse cholesterol transport, was associated with a 38% higher risk of chronic heart disease per standard deviation increase in methylation [14,18]. CpG sites combined in a methylation risk score explained up to 9% of the variance in TG [18].

A limitation of our study is that although we have replicated the *KREMEN2* signal (cg04580029) in adipose tissue, we did not have access to an obese population to further validate this signal, which should be considered in a future study.

In conclusion, our findings demonstrate novel associations between DNA methylation and TG levels, and propose some mechanisms that relate these associations with pathways affecting different diseases in humans that merit further investigation. These results could provide new insight in the mechanisms underlying CVD and contribute to new therapeutic interventions.

## 4. Materials and Methods

### 4.1. Participants

The discovery cohort of this study consists of 211 individuals from 73 families of the STANISLAS Family Study (SFS). The SFS is a 10-year longitudinal survey with 3 visits at 5-year intervals, involving 1,006 families from Vandoeuvre-lès-Nancy, France, first recruited between 1993 and 1995 [29]. It is a part of the Biological Resources Center “Interactions Gène-Environnement en Physiopathologie CardioVasculaire” (BRC-IGE-PCV, number BB-0033-00051). All subjects used for the study were of European-Caucasian origin, without the presence of serious and/or chronic disorders and personal history of CVD. The study protocols were approved by institutional ethics committees and all subjects gave written informed consent for their participation in the study. Population characteristics for the discovery cohort are compiled in Table 3.

The replication cohort consists of 662 adipose tissue samples collected in the MuTHER study. The MuTHER study includes 856 female European-descent individuals aged 59.12±94.44 years, recruited from the TwinsUK Adult Twin Registry. All the procedures followed were in accordance with the ethical standards of the St. Thomas’ Research Ethics Committee (REC reference 07/H0802/84) at St. Thomas’ Hospital in London, and all study subjects provided written informed consent.

### 4.2. Data Collection

For the SFS cohort, participant information including personal health, lifestyle, biological and clinical data was collected using appropriate, validated questionnaires and procedures [29,30]. Body mass index (BMI) was expressed as weight (kilograms) divided by height²·(meters)². Blood samples were collected between 8 and 9 a.m. after overnight fasting. DNA was extracted by the Miller technique [31] and was stored at –80 °C until further use.

For the MuTHER study, 8 mm punch biopsies were taken from a relatively photo-protected area adjacent and inferior to the umbilicus. Subcutaneous adipose tissue was carefully dissected from each biopsy, weighted and split into multiple pieces, and immediately stored in liquid nitrogen until analysis.

### 4.3. Biological Measurements

For SFS cohort, serum total cholesterol (TC) and serum TG levels were measured using standard enzymatic methods (Merck, Darmstadt, Germany), automated on AU5021 (Olympus; Merck). Serum HDL was measured by phosphotungstate precipitation on a Cobas-Mira (Roche, Basel, Switzerland), while LDL levels were calculated using the Friedewald formula [32]. Immunonephelometry was used for the detection of serum Apolipoprotein (Apo) B levels on Behring Nephelometer Analyser, with Behring reagents (Behring Diagnostics, Rueil-Malmaison, France) and serum ApoE was measured by turbidimetry [33].

### 4.4. DNA Methylation Assessment

#### 4.4.1. Discovery Cohort

Genome-wide DNA methylation profiling was performed by Infinium HumanMethylation450 BeadChip (Illumina, San Diego, CA, USA) [34], which performs two measurements for each CpG: a methylated and unmethylated intensity. DNA methylation is described as a β value, a ratio between methylated and unmethylated intensities. A detection *p*-value was generated for every CpG with minfi R package [35]. Poor quality probes were excluded from the analysis using a detection *p*-value cutoff >0.05. Furthermore, probes, missing in >5% of the samples were excluded from all samples. Based on these two criteria, 764 probes were removed from analysis. To avoid spurious associations, the genomic information of probes already annotated in HumanMethlyation450 annotation files (probes containing single-nucleotide polymorphism (SNP), sex chromosomes, and a single base extension (SBE) site) was excluded. Finally, probes containing cross reactive and target polymorphic CpGs [36] were excluded, leaving 373626 probes for statistical analysis.

Before the association analysis, background correction and normalization were carried out with Illumina background correction and SWAN [37] in the R package minfi, respectively. One individual from our cohort was excluded after quality control checks of methylation array data (outlier of plotted median of the methylated against unmethylated samples, with the signal below 10.5), leaving 210 individuals for analysis (Table 3).

#### 4.4.2. Replication Cohort

Methylation levels were estimated at 485,764 sites covering not only gene promoters but also several other genomic features in subcutaneous adipose tissue derived from the female twins as described in [38]. In brief, prior to bisulphite conversion, DNA samples were randomized, and exactly 700 ng of each DNA sample was taken for bisulphite conversion with the EZ-96 DNA Methylation Kit (Zymo Research, Irvine, CA, USA) according to the supplier’s protocol. Methylation profiling was performed with Illumina’s Infinium HumanMethylation450 BeadChip. Arrays were scanned with the IlluminaHiScan SQ scanner, and raw data were imported to the GenomeStudio v.2010.3 software with the methylation module 1.8.2 for the extraction of the image intensities.

### 4.5. Genotyping and Selection of Single-Nucleotide Polymorphisms (SNPs)

Genotyping was performed by the Infinium CoreExome Illumina assay. Significant TG associated SNPs were selected from the NHGRI-EBI GWAS catalog [39], located in the same chromosome as the significant methylation sites.

### 4.6. Statistical Analysis

Levels of TC, TG, LDL and HDL for discovery cohort were not normally distributed and were therefore transformed on the e-log scale. A linear mixed effects model was used to analyze the association between methylation levels at each probe and log-transformed phenotypes accounting for relatedness between individuals. The model used was including gender, age, BMI, family structure, smoking and individual blood cell counts (neutrophils, lymphocytes, monocytes, eosinophils and basophils) as covariates, and chip array as random effect. Q values were estimated for false discovery rates [40] and *q* values (<0.05) were considered for defining the statistical significance. False discovery rate (FDR) method [40] was used for the correction of the results for multiple testing and Bonferroni correction method (cut-off 3.35 × 10^−8^). The analysis of association of methylation values and the assessed phenotypes were performed by Package CpGassoc in R [41]. HumanMethlyation450 annotation files were used for annotating the probes and their corresponding genes.

Levels of TC in replication cohort followed the same statistical analysis procedure. The model used was adjusted for age, BMI, smoking status, family identification number, array batch and zygosity in a linear mixed effects model.

A linear mixed effects model with age, gender, BMI, and smoking as fixed effects, and methylation chip, and family structural as a random effect was carried out for the genetic association analysis using the statistical package lmerTest of R [42]. Significance cut off was set to 0.05/n where n is the number of significant TG associated SNPs in previous GWAS studies.

Deviation of Multiple Correlation Squared ρ2 From Constant (Random Model) of G*Power software was used in order to calculate statistical power [7], specifically the post hoc power analysis procedure. This procedure comprises a parameter analysis; it requires the test type (tails: one or two), effect size (H1 ρ2 and H0 ρ2), α error probability, total sample size, and number of predictors.

### 4.7. In Silico Analysis

The significant CpG sites were localized on the Human genome (GRCh38.p12) using Ensembl browser [43] and UCSC browser [44] as well as the establishment of regulatory feature activity of their respective promoters.

### 4.8. Data Access

Data is freely available upon request to the Biological Resources Centre IGE-PCV (http://www.u1122.inserm.fr/).

## Figures and Tables

**Table 1:** Association of methylation values with TG level.

CpG	Gene Name	Chromosome	Effect Size	SE	Unadjusted *p*-Value	FDR
cg08897188	*PRKAG2*	7q36.1	−2.80	0.47	1.39 × 10^−8^	0.049
cg04580029	*KREMEN2*	16p13.3	3.09	0.51	5.75 × 10^−9^	0.049

SE: Standard error, FDR: False discovery rate (<0.05).

**Table 2:** Replication of the associations of the 2 probes with TG levels in adipose tissue.

CpG	Gene Name	Effect Size	SE	*p*-Value
cg08897188	*PRKAG2*	−0.0008	0.0016	0.6265
cg04580029	*KREMEN2*	0.0084	0.0036	0.0196

SE: standard error.

**Table 3:** Populations characteristics of discovery cohort.

Population Characteristics	Total		Adults (116)		Children (95)		Male (105)		Female (106)	
	Mean	SD	Mean	SD	Mean	SD	Mean	SD	Mean	SD
**Age (years)**	28.17	14.83	40.48	7.53	13.15	2.58	27.09	15.14	29.24	14.37
**Body Mass Index (kg/m^2^)**	21.52	4.00	24.06	3.22	18.43	2.31	21.77	4.17	21.27	3.79
**Cholesterol (mmol/L)**	5.24	1.00	5.70	0.96	4.68	0.74	5.12	1.04	5.37	0.95
**Triglycerides (mmol/L)**	0.86	0.49	0.98	0.56	0.72	0.32	0.91	0.55	0.82	0.41
**High density lipoprotein (mmol/L)**	1.44	0.41	1.45	0.42	1.43	0.39	1.34	0.38	1.54	0.42
**Low density lipoprotein (mmol/L)**	3.63	0.97	4.06	0.96	3.11	0.68	3.59	1.02	3.67	0.91
